# Isotretinoin as a Possible Environmental Trigger to Autoimmunity in Genetically Susceptible Patients

**DOI:** 10.1155/2017/4207656

**Published:** 2017-03-26

**Authors:** Jocelyn Nugroho, Bahareh Schweiger

**Affiliations:** ^1^Western University of Health Sciences, 309 E 2nd St, Pomona, CA 91766, USA; ^2^Cedars-Sinai Medical Center, 8700 Beverly Blvd, Los Angeles, CA 90048, USA

## Abstract

*Introduction.* Isotretinoin is commonly used to treat cystic acne. Definitive mechanisms of action for isotretinoin are not known though despite many side effects having been documented. Various case reports have noted autoimmune diseases succeeding isotretinoin treatment.* Case Report.* A 16-year-old female presents with symptoms of tremors, lack of focus, sleeplessness, emotional liability, bulging eyes, loose stools, heat intolerance, and missed menstrual periods. Symptoms manifested shortly after the patient finished a course of oral isotretinoin treatment for acne. Physical exam showed resting tremors, bilateral proptosis, hyperactivity, and rapid speech. A diagnosis of Graves' Disease was made by correlating symptoms, physical exam findings, ultrasound, and positive family history of autoimmune thyroid disease.* Conclusion.* Emergence of autoimmune thyroid diseases depends upon genetic predisposition and environmental triggers. Mechanism of action for isotretinoin is not known but the drug may play a role in triggering autoimmunity in genetically susceptible individuals.

## 1. Introduction

Isotretinoin, also known as 13-cis retinoic acid, is commonly used as a potent remedy for cystic acne. The exact mechanism of action for isotretinoin, however, is not completely understood [1, 2]. It is postulated that isotretinoin achieves therapeutic effects by altering the cell cycle, specifically cellular differentiation, in addition to modifying cell survival and cell death [2]. Side effects of isotretinoin range from fatigue to depression, muscle aches, constipation, teratogenicity, and, more commonly, mucocutaneous side effects leading to irritable bowel syndrome [2, 3]. Various case reports have also reported emergence of autoimmune disease such as diabetes, autoimmune hepatitis, Guillain-Barre syndrome, and thyroiditis shortly after completing a regime of isotretinoin or during last week of treatment [4–8]. The case presented below describes a patient with a positive family history of autoimmune thyroid disease who develops Graves' Disease shortly after completing a course of isotretinoin treatment for cystic acne.

## 2. Case Presentation

A 16-year-old female was referred to our Pediatric Endocrinology Clinic in October 2016 after receiving abnormal thyroid function test results. The patient had recently completed her fourth course of oral isotretinoin treatment for cystic acne in August. Soon after treatment cessation, she began to experience symptoms of tremors, lack of focus, inability to fall asleep, emotional liability, bulging eyes, loose stools, heat intolerance, and missed menstrual periods during her last two cycles.

Family history was positive for thyroid disorders; a maternal aunt was diagnosed with hyperthyroidism and a cousin with hypothyroidism. During physical examination, the patient was hyperactive with rapid speech and displayed a moderate degree of proptosis bilateral with an unblinking stare. On palpation, the thyroid was diffusely enlarged, firm, and homogenous. A resting tremor was also observed.

Thyroid function tests were repeated and revealed a TSH < .02 (normal range: 0.45–4.50), total T4 of 16.5 (normal range: 4.5–12.0), total T3 of 5.1 (normal range: 2.0–4.4), and TSI of 358% (normal range: <140%). Thyroid ultrasound was also performed and showed a heterogeneous thyroid gland with increased vascular flow. Symptoms, physical exam, thyroid function test, and thyroid ultrasound were all consistent with a diagnosis of autoimmune hyperthyroidism, also known as Graves' Disease.

The patient was started on methimazole 20 mg daily as well as atenolol 25 mg daily and asked to follow up in one month. Isotretinoin was not used during time of hyperthyroid treatment.

At one-month follow-up, the patient stated that she was sleeping better and feeling less shaky, although she did admit to still having difficulty in concentrating as well as hair loss. Physical exam revealed bilateral proptosis with mild improvement, hyperactivity, rapid speech, resting tremor, clubbing of fingers, and an enlarged thyroid.

Her methimazole regime was reduced to 15 mg daily, and atenolol 25 mg daily was to be ceased after 7 days with blood pressure monitoring thereafter. The patient was asked to return for follow-up in 4 months.

## 3. Discussion

Autoimmunity is thought to emerge from an interplay of genetic and environmental factors [9]. Patients who are genetically susceptible to developing autoimmunity may come in contact with environmental triggers, which in turn weaken the tolerance of the patient causing autoimmune disease [10]. Environmental triggers are thought to include many factors like drugs, toxins, chemicals, stress, diet, and viral infections [9, 10]. In the case presented above, the patient has a positive family history of autoimmune induced thyroid disease, thereby indicating genetic susceptibility. In this specific patient's case, it is possible that the environmental trigger that initiated the breakdown of immune tolerance was her recent course of isotretinoin treatment.

Studies have shown that isotretinoin treatment alters pituitary hormone levels and downstream axis hormones [11]. In studies focusing on thyroid hormone levels following isotretinoin treatment, it has been noted that, by the end of the treatment course, patient's TSH levels are elevated, while free T3 and T4 levels are decreased [3, 12]. It is possible that alternations of TSH levels could be due to isotretinoin effects on nuclear retinoid X receptors (RXR), which dimerizes with nuclear retinoid acid receptors (RAR) and nuclear thyroid receptors [2, 13]. Although isotretinoin itself does not directly interact with RXR, it is thought that it is converted intracellularly to an active form, thereby altering expression of TSH and other hormones [2, 3].

Several case studies have noted the development of autoimmune thyroiditis in patients following or within the last few weeks of isotretinoin treatment. For example, in 2012, Gursoy et al. reported a case describing a 19-year-old male patient presenting with symptoms and positive laboratory findings for autoimmune hyperthyroid thyroiditis and ocular myasthenia gravis. The patient had no family history of autoimmune or neuromuscular disease [8]. On a separate note, in 2014, Guerouaz et al. reported a case on 2-year-old twin brothers who were treated with isotretinoin for inflammatory acne conglobate. The twins developed autoimmune thyroiditis [7]. In both these cases, either patients had a negative family history of autoimmune thyroid disease or no family history was recorded.

Currently, the exact mechanism by which isotretinoin acts is unknown, let alone how it induces autoimmune thyroiditis. That said, it is possible that isotretinoin mediated alterations to nuclear RXR dimerization to RAR and nuclear thyroid receptors may provide a catalyst for promoting autoimmunity [2, 13].

Further studies on patients with a known familial predisposition to autoimmune thyroid diseases and the likelihood of patients developing autoimmune thyroiditis on isotretinoin treatment may provide further insight into the degree to which isotretinoin acts as an environmental trigger for autoimmunity.

## 4. Conclusion

This case describes a patient with positive family history of autoimmune hyperthyroidism and hypothyroidism who developed symptoms of autoimmune hyperthyroidism after completing a course of isotretinoin for cystic acne. Further studies should be done to determine the extent to which isotretinoin acts as an environmental trigger to autoimmune thyroiditis. Until then, clinicians should be more cautious when prescribing isotretinoin to patients with a known family history of autoimmune disease.
